# Dielectric Breakdown and Post-Breakdown Dissolution of Si/SiO_2_ Cathodes in Acidic Aqueous Electrochemical Environment

**DOI:** 10.1038/s41598-018-20247-x

**Published:** 2018-01-30

**Authors:** Jeongse Yun, Yun-Bin Cho, Woohyuk Jang, Jae Gyeong Lee, Samuel Jaeho Shin, Seok Hee Han, Youngmi Lee, Taek Dong Chung

**Affiliations:** 1 Department of Chemistry, Seoul National University, Seoul, 08826 Republic of Korea; 2 Department of Chemistry and Nano Science, Ewha Womans University, Seoul, 03760 Republic of Korea; 3 Advanced Institutes of Convergence Technology, Suwon-si, Gyeonggi-do 16229 Republic of Korea

## Abstract

Understanding the conducting mechanisms of dielectric materials under various conditions is of increasing importance. Here, we report the dielectric breakdown (DB) and post-breakdown mechanism of Si/SiO_2_, a widely used semiconductor and dielectric, in an acidic aqueous electrochemical environment. Cathodic breakdown was found to generate conduction spots on the Si/SiO_2_ surface. Using scanning electrochemical microscopy (SECM), the size and number of conduction spots are confirmed to increase from nanometer to micrometer scale during the application of negative voltage. The morphologies of these conduction spots reveal locally recessed inverted-pyramidal structures with exposed Si{111} sidewalls. The pits generation preceded by DB is considered to occur via cathodic dissolution of Si and exfoliation of SiO_2_ that are induced by local pH increases due to the hydrogen evolution reaction (HER) at the conduction spots. The HER at the conduction spots is more sluggish due to strongly hydrogen-terminated Si{111} surfaces.

## Introduction

The properties of dielectric materials have been studied intensively with the rapid development of integrated circuit technology. Most studies focused on events within the solid-state structures, such as metal-oxide-metal or metal-oxide-semiconductor systems^1,2^. Among those events, electrical conduction through the thin dielectric film is a key phenomenon related to the reliability of electronic devices. Percolation model is one of the models explaining electrical conduction of dielectric materials in solid state physics^3^. According to this model, point defects are randomly created in the dielectric film when an electric field is applied. With increasing defect concentration, clusters of defects within tunneling distance of one another connect both sides of the film facilitating electron flow through the film; this is referred to as “dielectric breakdown (DB)” and results in a sudden change in electrical behavior as a large current begins to flow through the film.

Electrical conduction of thin dielectric films has recently attracted attention because of the growing interest in their applications to resistive memory devices^4^, nanopore generation^5,6^, and photoelectrochemical energy conversion^7^. For electrochemical systems which employ semiconductor, dielectric films as passivation layers should be considered to prevent the semiconductor from directly contacting to the electrolyte or metal^7–9^. Although studies on dielectric materials in the solid state have provided significant amounts of information, there are only a limited number of studies about these materials when immersed in solution. Unlike solid phase conductive or semiconducting materials, solution phase has quantified energy states determined by the reduction-oxidation reactions that occur on the electrode surfaces. Furthermore, in an aqueous environment in particular, various species such as protons and water molecules can chemically or electrochemically affect dielectric films and their underlying solid materials^10–13^. It is therefore anticipated that DB kinetics will be affected by various chemical species in solution.

Damage caused by DB in contact with solution would differ from that occurring in solid state device. Permanent damage of metal oxide semiconductor capacitor (MOSCAP) device takes place in several ways: melting or cratering of gate metal or oxide and epitaxial growth of semiconductor have been reported^3,14–16^. Joule-heating caused by large current density through local defect rich region is the major reason of the post-breakdown damages. On the other hand, in electrolyte-oxide-semiconductor or electrolyte-oxide-metal capacitor, DB is expected to be accompanied by local corrosion of underlying semiconductor or metal as a post-breakdown damage, which has been considered to initiate within the range of nanometers and micrometers^17^.

Scanning electrochemical microscopy (SECM) is a useful technique to locally probe microscopic processes on electrode surfaces within a microscale distance in an electrochemical cell. Using a probe microelectrode, SECM allows surface characterization with a spatial resolution of micrometer level or below; and thus is expected to be appropriate for the observation of DB and following post-breakdown processes^18^. To the best of our knowledge, there has been no report which investigates the localized DB process and successive events occur on dielectric film in solution. In this study, SECM will give direct and microscopic information about DB and post-breakdown change of SiO_2_ in an aqueous environment.

This study reports on the electrochemical DB phenomenon and post-breakdown change of a thermal SiO_2_ film on highly doped n-type Si (Si/SiO_2_) in a weakly acidic 0.1 M phosphate-buffered solution (PBS, pH 3). A 6-nm-thick thermal SiO_2_ film was prepared by the dry oxidation of a Si wafer in a dry oxygen environment at 850 °C. The geometric area of the exposed SiO_2_ was fixed by a photoresist film. The electrochemical characteristics of Si/SiO_2_ experienced DB and post-breakdown were examined using conventional electroanalytical techniques and SECM. The electrode surface after DB and post-breakdown was analyzed by scanning electron microscopy (SEM) and transmission electron microscopy (TEM).

## Results and Discussion

**Figure 1:**
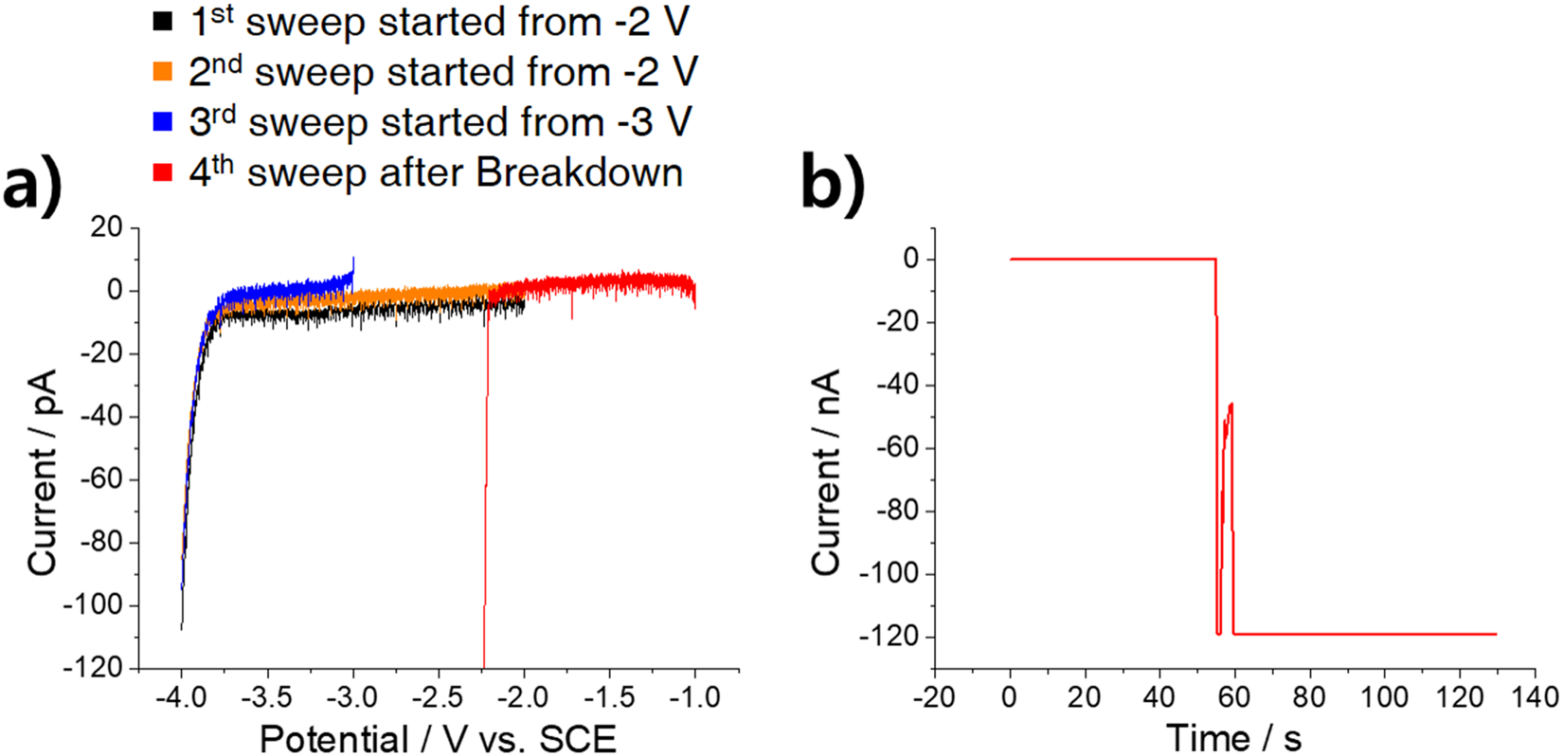
Characteristic electrochemical behavior of a highly doped n-type Si electrode with a 6-nm-thick SiO_2_ film in 0.1 M PBS (pH 3). The exposed electrode was 5 × 5 μm^2^ in size. All potentials are referenced to SCE. (**a**) Consecutive linear sweep voltammograms (20 mV s^−1^). The first (black) and the second (orange) voltammograms begin at −2 V, while the third (blue) voltammogram begins at −3 V. The fourth voltammogram (red) was obtained after breakdown, as shown in (**b**). (**b**) Chronoamperogram conducted between the third and the fourth voltammograms in which the potential was held at −4 V. The current exceeded the measurement limit after breakdown, which occurred after 55 s of elapsed time.

Representative current-voltage characteristics of a Si/SiO_2_/buffer electrochemical system are shown in Fig. 1a. During the cathodic sweep, the current increases gradually above a potential of −3.7 V (black curve). A subsequent cathodic scan within the same potential range provides a reproducible current-voltage curve (orange curve). To rule out any possible recovery of dielectric properties at −2 V, a subsequent scan starting at −3 V was performed (blue curve), which provided a similar voltammogram to the previous ones. This reveals that the reproducible curves are not the result of the electrical regeneration of the dielectric film and shows that DB has not yet occurred. The gradual current increase is attributed to charge injection into the oxide film, which results from the generation of defects in dielectric materials prior to DB^5^. Although the exact chemical structure of the defects is not fully understood, it is considered that hydrogen-related defect plays significant role in DB. Hydrogen bridge defect having a structure of Si-H-Si not only provides electron trap for SILC but also catalyzes reduction of SiO_2_ resulting in oxygen vacancy breaking the stoichiometry of the oxide^2–4^. The current-voltage relationship changed significantly after a five- or six-orders-of-magnitude larger current flowed whether by constant voltage stress (−4 V) (Fig. 1b) or by a current-voltage sweep to further negative potential (data not shown), implying that a permanent chemical or physical change had occurred on the Si/SiO_2_ electrode surface (red curve in Fig. 1a). This change cannot be explained by the exfoliation of the oxide from the underlying conductive Si because the linear-sweep voltammogram acquired after the breakdown is very different from that obtained with bare Si directly exposed to PBS solution after HF chemical etching (Figure S1). Although the hydrogen evolution reaction (HER) begins to appear at a mild overpotential (−0.7 V) on the bare Si electrode, the HER on the Si/SiO_2_ electrode after breakdown began at around −2.3 V. The sluggish HER on Si/SiO_2_ is discussed below.

Under constant voltage stress, time-dependent dielectric breakdown (TDDB) of the film occurs (Fig. 1b). Before DB, a small leakage current, known as the “stress-induced leakage current” (SILC) was observed (Figure S2), resulting from an increase in the defect concentration. After a certain amount of time, which is referred to as breakdown time (*t*_bd_), current suddenly increases from sub-nA to μA, indicating DB (Figure S3). After this sudden rise, the current was observed to increase irregularly. *t*_bd_ varied widely from a few seconds to several hundreds of seconds. According to the percolation model, the large deviation of *t*_bd_ is general characteristics of thin dielectric films^3,19^.

**Figure 2:**
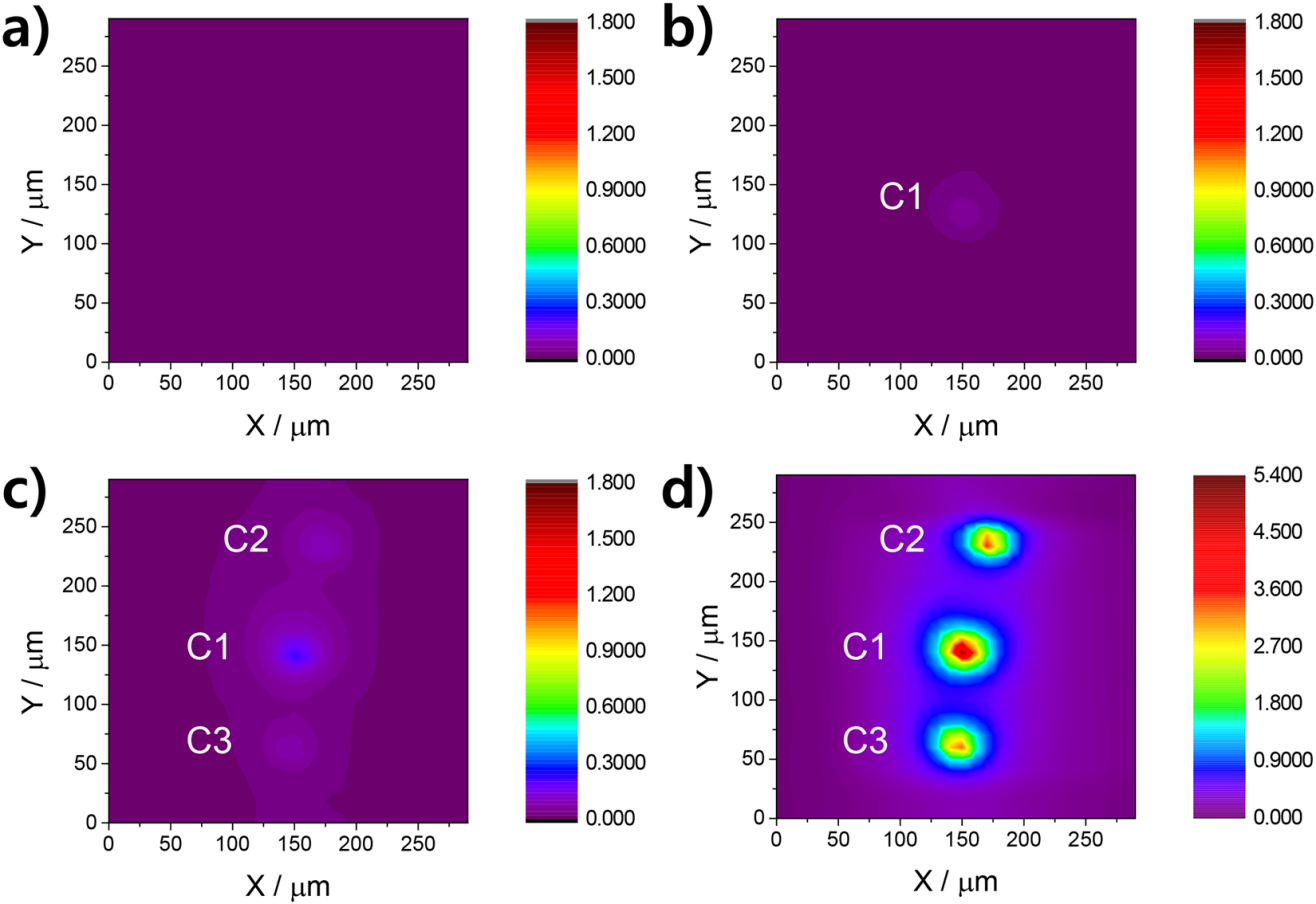
SECM images of a 200 × 200 μm^2^ Si/SiO_2_ substrate electrode obtained in SG-TC mode. The [Ru(NH_3_)_6_]^2+^ oxidation current of a tip with a potential (*E*_tip_) of +0.1 V in 10 mM [Ru(NH_3_)_6_]Cl_3_/0.1 M PBS (pH 3) was monitored while −1 V was applied to the Si/SiO_2_ substrate. The tip-to-substrate distance was 10 μm and the scan rate was 50 μm s^−1^. The units of tip current (color contours) are nA. (**a**) Before breakdown and (**b)** at ~10 s following a sudden increase in current when −4 V was applied to the Si/SiO_2_ substrate in 0.1 M PBS (pH 3). After additional (**c**) 750 s and (**d**) 950 s of −4 V applied to the Si/SiO_2_ substrate shown in (**b**) in 0.1 M PBS.

According to the solid-electronics literature, the DB of various oxide materials is generally known to occur at relatively weak regions of their oxide structures^20^. The weak regions would be defect-rich or thin parts of dielectric film, although the exact physical and chemical features of the regions are still unclear yet. The SECM results in this work also reveal the occurrence of a similar localized breakdown to that studied in the solid phase. SEM and SECM images of a 200 × 200 μm^2^ Si/SiO_2_ substrate electrode obtained in normal feedback mode confirm the presence of a smooth, physical-defect-free substrate surface (Figure S4). SECM substrate-generation tip-collection (SG-TC) images over the 200 × 200 μm^2^ area were obtained in 10 mM [Ru(NH_3_)_6_]Cl_3_/PBS solution (pH 3) before and after DB (Fig. 2). The images display tip currents (at *E*_tip_ = + 0.1 V) induced by the collection and re-oxidation of the [Ru(NH_3_)_6_]^2+^ generated at the substrate (at *E*_sub_ = −1 V). Figure 2a verifies the absence of pinholes on the oxide over the measured area, while Fig. 2b displays a local Si/SiO_2_ conduction spot generated within ~10 s after a sudden current increase at *E*_sub_ = −4 V, referred to as “C1”, where a large tip current was observed to flow. The greatest C1 tip current measured was ~55.5 pA (Fig. 2b). A further constant voltage stress following DB resulted in the increased number of conduction spots as well as the current increase at the previously generated conduction spot: 0.224 nA for C1 and two new conduction spots (C2 and C3 which have 82.6 pA and 0.101 nA, respectively) appeared after additional 750 s of −4 V imposition (Fig. 2c). The following 200-s application of −4 V caused further increases in tip collection currents: The greatest current reached to 5.02 nA for C1, 3.30 nA for C2 and 3.50 nA for C3 (Fig. 2d). According to these results, it is inferred that post-breakdown damage enlarges conduction spots. Figure S5a shows the resulting SEM images of the same Si/SiO_2_ substrate as shown in Fig. 2d. It shows that a constant potential supply for additional 950 s subsequent to DB generates recessed conduction spots where the surface oxide is removed. The structures of final conduction spots are seemingly developed via the connection of two or more neighboring recessed conduction spots of rectangular projection geometry (Figure S5b). The projected surface area of each recessed structure varies from 4.268 μm^2^ to 25.16 μm^2^.

Simulation using the COMSOL Multiphysics v. 5.2 software (COMSOL, Inc., Burlington, MA) reveals that a 10-μm diameter tip electrode can collect ~56% of the products generated from disk-shaped sources (*ϕ* 100 nm ~5 μm) over distances of 10 μm (not shown). Simply assuming that the conduction spot is a disk-type ultramicroelectrode (UME), its size can be calculated from the tip current using equation (1):

1 $${i}_{\mathrm{lim}}=4nFDCa\ldots \ldots \ldots $$

where *i*_*lim*_ is the measured limiting current, *n* is the number of electrons, *F* is the Faraday constant, *D* is the diffusion coefficient of [Ru(NH_3_)_6_]^2+^ (9.12 × 10^−6^ cm^2^ s^−1^, calculated from the literatures^21,22^), *C* is the concentration of [Ru(NH_3_)_6_]^3+^, and *a* is the radius of the electrode.

The estimated sizes of C1, C2 and C3 from the local maximum of tip currents in Fig. 2d are 5.094 μm, 3.347 μm and 3.552 μm in diameter, respectively, assuming the circular shape. As shown in Figure S5c, the actual conduction spots have quite similar dimensions to the corresponding disks estimated from the SECM tip currents. This suggests that the strategy of utilizing the highest tip current with the assumption of disk shaped conduction spot is acceptable to estimate the approximate sizes of recessed conduction spots. Figure S6 shows the SEM images of conduction spots created at the earlier stage after a current surge with continuous voltage application of *E*_sub_ = −4 V in 0.1 M PBS. Interestingly, with a constant potential supply (−4 V) for ~10 s and ~100 s subsequent to DB on Si/SiO_2_, the recessed structures with a rectangular projection surfaces appeared while their surface oxides still remained partially over Si. Due to the partial coverage of recessed structures with the surface oxides, the sizes estimated from the measured SECM tip currents were much smaller (2894 nm^2^, 923.5 nm^2^ and 0.5917 μm^2^ for Figures S6a, S6b and S6c, respectively) than the actual recessed regions observed in the SEM images (2.674 μm^2^, 2.305 μm^2^ and 10.11 μm^2^ for Figures S6a, S6b and S6c, respectively).

**Figure 3:**
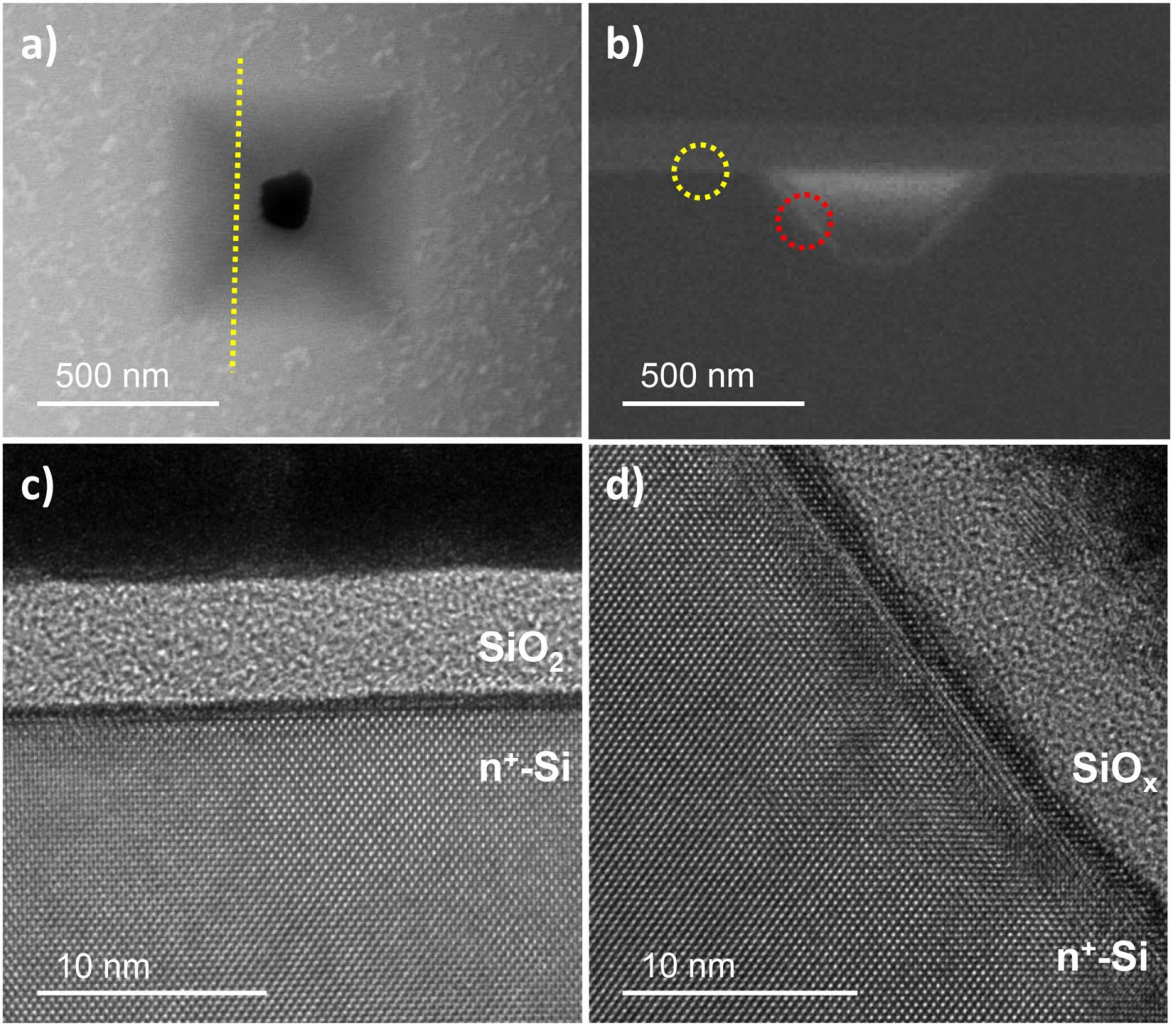
Representative SEM and TEM images of the inverted-pyramidal structure resulting from cathodic breakdown and post-breakdown etching. SEM images of (**a**) the top-view and (**b**) a cross-sectional side view along the trajectory indicated by the yellow dotted line in (**a**). Cross-sectional TEM images of (**c**) the undamaged Si{100} surface (yellow-dotted circle in (**b**)) and (**d**) the sidewall of the inverted-pyramidal structure that displays the stepped Si{111} surface (red-dotted circle in (**b**)).

The morphology of the recessed structures created after DB is found to be an inverted pyramid shape as shown in Fig. 3. Before DB, any physical damage was not observed on the surface oxide of Si/SiO_2_ although it had been under constant voltage stress at −4 V for 250 s (not shown). Thus, it is inferred that inverted pyramid structures appeared as a post-breakdown phenomenon. According to Fig. 3b, The angle between the sidewalls and the {100} surface of the wafer is 55°, suggesting that the newly generated crystalline surfaces are Si{111}^23^. TEM analysis reveals that the Si{111} sidewall is atomically rough with multiple steps (Fig. 3d), whereas the undamaged Si{100} surface is atomically smooth (Fig. 3c).

Post-breakdown damage is often explained by Joule-heating of the local conduction path within the oxide because a large electrical current flows along a very narrow percolation path^3,5^. Various types of post-breakdown damage were reported such as epitaxial growth of silicon and gate metal burning in a solid state device. Nanoscale pore can be created when dielectric film has a direct contact to aqueous electrolyte^24,25^. This nanoscale pore generation is attributed to dissolution of a percolation path in the dielectric film where stoichiometry is changed due to DB^26,27^. In our experiment, the inverted-pyramidal structure is expected to appear after dissolution of percolation path and appears to be created by the dissolution reaction of Si, as indicated by the flat-etched crystalline surface. This dissolution hypothesis is supported by the partially covered oxide film in the dissolved region (Figures S6 and S7). According to Liu *et al*., cathodic dissolution occurs under external stresses of tens to hundreds of volts in a humid atmosphere when the cathode is much smaller than the anode^28^. They suggest that cathodic dissolution is facilitated by pH increases resulting from HER near the cathode. The generation of the inverted pyramid (Fig. 3) in our study could be explained similarly: the local pH increase at the narrow conduction spot due to nearby HER may trigger dissolution of the underlying Si. It is no wonder that a larger conduction region leads to more HER. Therefore, the thin oxide film covering the conduction region is unable to resist rapid HER, and is then exfoliated.

As mentioned above, the HER is suppressed at the Si/SiO_2_ conduction spots and requires larger overpotentials than that at a Si{100} wafer. This is ascribed to the stable hydrogen-terminated surface of the Si{111} sidewall generated at the conduction spot; hydrogen atoms terminate the Si surface at the cathodic potential^29^. Among the crystalline surfaces of Si, the {111} surface forms the most stable hydrogen terminations^30^. As a consequence, due to strong hydrogen adsorption on the Si{111} surface, HER following DB requires a larger overpotential than at other crystalline surfaces and is therefore more sluggish.

**Figure 4:**
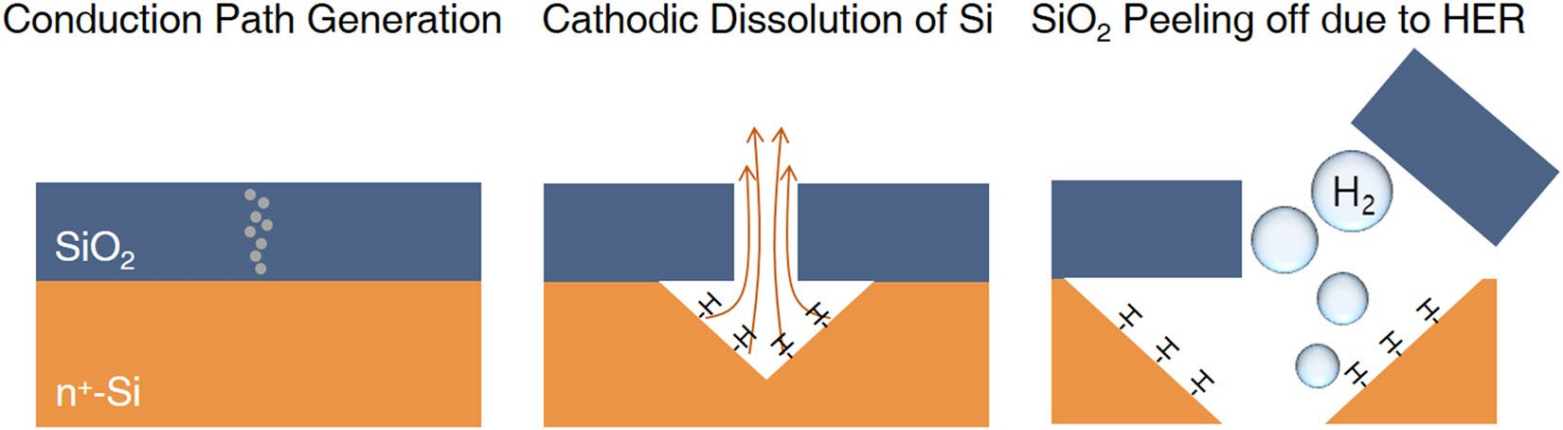
Schematic diagram of mechanism for the breakdown and post-breakdown of Si/SiO_2_ under acidic conditions.

Based on our findings, we propose a mechanism for the DB and post-breakdown of Si/SiO_2_ under acidic conditions, as shown in Fig. 4. First, defects generated within the SiO_2_ film by the applied cathodic potential create conduction spots through percolation paths that connect Si to the solution; this is referred to as “DB” and these paths are dissolved out of the oxide. Secondly, the cathodic dissolution of Si occurs as post-breakdown dissolution since the HER increases the local pH at the narrow conduction spot; meanwhile, the Si{111} surface is continuously exposed and terminated by hydrogen. Finally, vigorous HER exfoliates the covering SiO_2_ film, leading to an inverted-pyramid-shaped structure on the Si/SiO_2_.

## Conclusions

In summary, DB and post-breakdown dissolution of Si/SiO_2_ under aqueous acidic conditions was examined in this study. When a strong negative potential (−4 V) is applied to the Si/SiO_2_, the 6-nm thick SiO_2_ film loses its dielectric properties and conduction spots are produced within several hundreds of seconds. The sizes of these conduction spots grow into micrometer scale during the post-breakdown process since HER leads to cathodic dissolution and SiO_2_-layer stripping at the conduction spots. At the same time, the number of conduction spots increases. The DB and the subsequent cathodic dissolution cause permanent structural changes to the electrode, resulting in inverted-pyramid-shaped structures. A newly exposed Si{111} crystalline surface is generated through cathodic dissolution, and this orientation results in rather suppressed HER through the formation of stable hydrogen terminated surface. Microscopic characterization of the local conduction spots on Si/SiO_2_ following DB and post-breakdown in aqueous solution provides a fundamental understanding of the Si/SiO_2_ breakdown mechanism and new insight into the conduction of dielectric materials in an aqueous electrochemical environment.

## Materials and Methods

### Materials

[Ru(NH_3_)_6_]Cl_3_ and Kimble microcapillary pipettes were from Sigma-Aldrich (St. Louis, MO). Phosphate buffer solution (PBS) was made by mixing NaH_2_PO_4_ and Na_2_HPO_4_ ($\ge$99.9%) from Sigma-Aldrich. Pt microwire (10-μm diameter) was purchased from Nilaco (The Nilaco Corporation, Tokyo Ginza). Highly doped n-type silicon wafer (arsenic-doped, <100>-oriented) with a resistivity as low as 0.005 Ω cm was obtained from STC (Japan). AZ9260 photoresist was purchased from Merck (USA).

### Preparation of thermal oxidized Si/SiO_2_

The 6-nm thick thermal SiO_2_ film was prepared by the dry oxidation of the Si wafer in oxygen environment at 850 °C. After cleaning with a mixture of H_2_SO_4_ and H_2_O_2_, the native oxide was stripped by HF dipping, and a 20-nm-thick thermal SiO_2_ layer was produced at 850 °C in a furnace with dry O_2_ blowing. Next, the 20-nm-thick thermal oxide layer was stripped again by HF dipping. After repetitive cleaning, 6-nm-thick thermal SiO_2_ layers were formed at 850 °C in a furnace with dry O_2_ blowing.

### Photoresist coating and lithography on Si/SiO_2_

To fix the exposed SiO_2_ area and reduce unwanted pinholes in the oxide film, photoresist coating and lithography were performed on the prepared Si/SiO_2_ as follows. The whole wafer of Si/SiO_2_ was cleaned by hot piranha solution (the 3:1 mixture of sulfuric acid and 30% hydrogen peroxide) for 15 min. After being washed with excess amount of deionized water, the wafer was placed on 200 °C hot plate for 15 min to remove water. AZ9260 photoresist was spin coated on the wafer at 6000 rpm for 30 s. Soft bake was conducted at 110 °C for 1.5 min. Then the wafer was aligned under a pattern-designed chromium mask (5 × 5 μm^2^ or 200 × 200 μm^2^ square array), and exposed to UV lamp. Developing was conducted by immersing the wafer into AZ400K developer (Merck, USAs) for 2 min. After judging whether the wafer was well developed by optical microscope, hard bake was conducted at 200 °C for 15 min.

### Electrochemical characterization

To minimize mechanical stress, the whole wafer without a dicing process was used for all the electrochemical experiments. For the electrical connection to Si/SiO_2_, the oxide layer on the back of the silicon wafer was removed by scratching with a diamond point pen by ~1 cm^2^ and casting a droplet of 48% hydrofluoric acid solution. This area was covered by gallium-indium eutectic (≥99.99% trace metals basis from Sigma-Aldrich) and then attached by ~10-cm-long conductive adhesive tape. The tape was connected to the working electrode cable of the electrochemical analyzer. Tens-of-microliters of PBS solution were dropped onto an exposed SiO_2_ area to form an electrochemical cell (Figure S8). Electrochemical characterization via a conventional electroanalytical technique was performed using an electrochemical analyzer (CHI 750, CH Instrument). Pt wire and a saturated calomel reference electrode (SCE) with a saturated-KNO_3_ double junction were employed as the counter and reference electrodes, respectively. All potentials in this paper are referenced to SCE. Linear sweep voltammetry (LSV) and chronoamperometry (CA) at a constant applied potential (−4 V) were carried out in 0.1 M PBS solution (pH 3) to see characteristic electrochemical behavior of Si/SiO_2_ during DB and post-breakdown.

### Tip electrode fabrication

A tip electrode was prepared by sealing Pt microwire (10 μm in dia.) under vacuum in glass capillary (outer dia. = 1.5 mm, inner dia. = 0.5 mm) followed by vertical polishing to expose a Pt microdisk at the end plane, as previously reported^18^. The glass sheath of the prepared 10-μm diameter Pt disk ultramicroelectrode (UME) was grinded to make its RG = 4–6. RG is the ratio of the overall tip electrode radius including a glass sheath to the Pt disk radius.

### SECM measurements

Electrochemical measurements using SECM were performed in a four-electrode setup using an electrochemical analyzer (CHI 920 C SECM) with a Pt wire and a saturated calomel reference electrode (SCE) with saturated-KNO_3_ double junction as the counter electrode and reference electrode, respectively. A tip was approached to the vertical distance of 10 μm above Si/SiO_2_ substrate in 10 mM [Ru(NH_3_)_6_]Cl_3_/PBS solution (pH 3). To find the location of 200 × 200 μm^2^ Si/SiO_2_ substrate electrode, the tip was scanned in x- and y-directions. To confirm the location of the substrate electrode and the absence of defects, a SECM image of 200 × 200 μm^2^ Si/SiO_2_ substrate electrode was obtained in a normal feedback mode, monitoring the [Ru(NH_3_)_6_]^3+^ reduction current at the tip with the tip potential (*E*_tip_) held at −0.5 V (vs. SCE) in a fresh 10 mM [Ru(NH_3_)_6_]Cl_3_/0.1 M PBS (pH 3) without applying any potential to the Si/SiO_2_ substrate. Next, SECM images of the Si/SiO_2_ substrate were obtained in substrate generation-tip collection (SG-TC) mode before and after breakdown induced with a continuous −4 V application to the substrate. In fact, −1 V, sufficient for [Ru(NH_3_)_6_]^3+^ reduction, was applied to the substrate; and the tip current with *E*_tip_ =  + 0.1 V, responding to [Ru(NH_3_)_6_]^2+^ oxidation, was monitored concurrently while the tip was scanned over the substrate in x- and y-directions in a fresh 10 mM [Ru(NH_3_)_6_]Cl_3_/0.1 M PBS (pH 3). This process was repeated after further breakdown in 0.1 M PBS (pH 3). For all SECM measurements, the probe scan rate was 50 μm s^−1^ (increment distance = 10 μm, increase time = 0.2 s).

### SEM and TEM measurements

The morphology of the Si/SiO_2_ electrode was examined by field-emission scanning electron microscopy (FE-SEM, Hitachi S-4300) operated at 15 kV. Pt coating on the electrode was performed before the FE-SEM measurement. The TEM sample was prepared by focused ion beam (FIB) gun with FE-SEM (Helios 650, FEI, USA). The inverted pyramid structure was found by FE-SEM, and then Pt and carbon were sequentially deposited on the inverted pyramid for sample protection. The TEM sampling area was selected whose cross section to be perpendicular to the surface and the flat of the wafer simultaneously. The Cs-STEM measurement was performed with JEM-ARM200F (cold field emission type, JEOL Ltd, Japan) operated at 200 kV.

### Data Availability

All data supporting the findings of this study are included in this article and its supplementary information file.

## Electronic supplementary material

Supplementary Material
