# Supplementary material for: Dielectric Breakdown and Post-Breakdown Dissolution of Si/SiO2 Cathodes in Acidic Aqueous Electrochemical Environment
Source: Sci Rep. 2018 Jan 30;8:1911. doi: 10.1038/s41598-018-20247-x (PMC5789982; doi:10.1038/s41598-018-20247-x)
Supplement: Supplementary file 1 — Supplementary Material [file 41598_2018_20247_MOESM1_ESM.pdf]

*Supplementary material*

# Dielectric Breakdown and Post-Breakdown Dissolution of Si/$SiO_2$ Cathodes in Acidic Aqueous Electrochemical Environment

Jeongse Yun[1], Yun-Bin Cho[2], Woohyuk Jang[1], Jae Gyeong Lee[1], Samuel Jaeho Shin[1], Seok Hee Han[1], Youngmi Lee[2,*], Taek Dong Chung[1,3,*]

[1] Department of Chemistry, Seoul National University, Seoul, 08826, Republic of Korea.

[2] Department of Chemistry and Nano Science, Ewha Womans University, Seoul, 03760, Republic of Korea.

[3] Advanced Institutes of Convergence Technology, Suwon-si, Gyeonggi-do, 16229, Republic of Korea.

J.Y. and Y.-B.C. contributed equally to this work.

[*] Correspondence and requests for materials should be addressed to T.D.C. (email: tdchung@snu.ac.kr) or Y. L. (email: youngmilee@ewha.ac.kr)

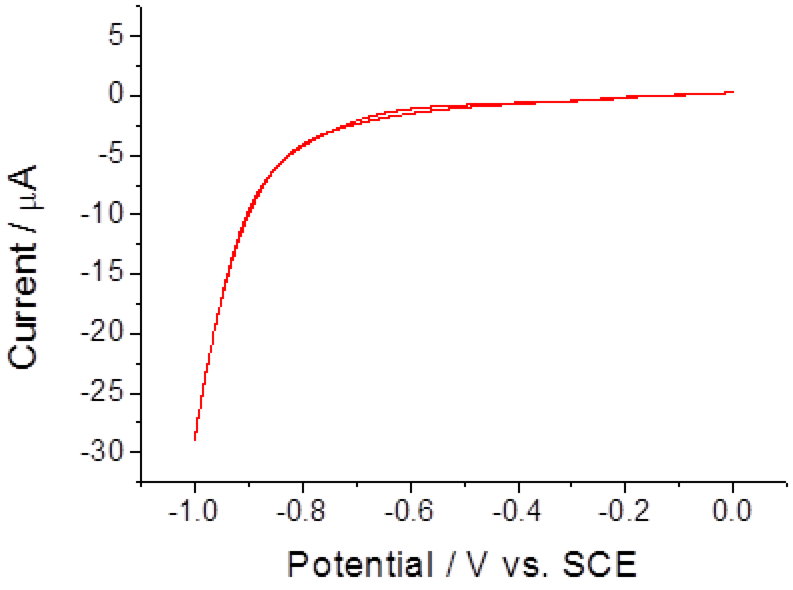


**Figure S1.** A cyclic voltammogram of a HF-etched Si/$SiO_2$ obtained in 0.1 M PBS (pH 3) at a scan rate of 20 mV $s^{-1}$.

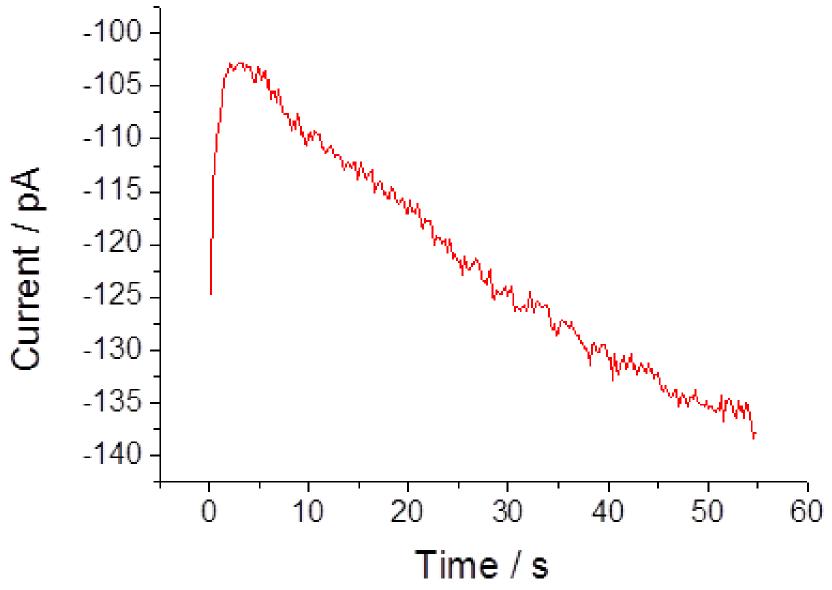


**Figure S2.** A current-time response of a 5×5 μm$^2$ Si/$SiO_2$ applied with a potential of −4 V (vs. SCE) as a function of time in 0.1 M PBS (pH 3). This is the magnified image of Figure 1b until 55 s.

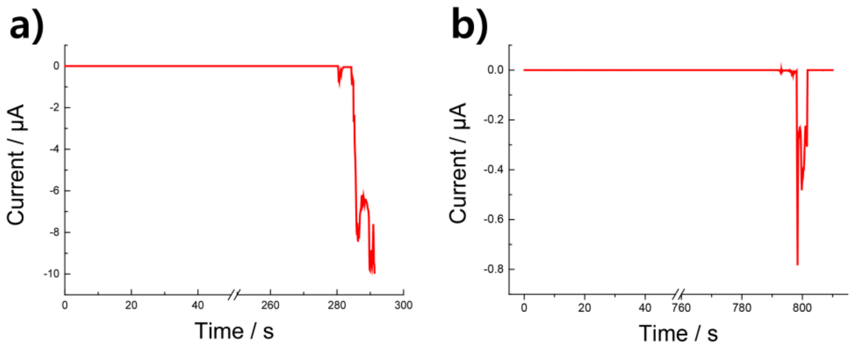


**Figure S3.** A current-time response of a 200×200 μm$^2$ Si/SiO$_2$ applied with a potential of −4 V (vs. SCE) as a function of time in 0.1 M PBS (pH 3). A temporary decrease after a sharp increase in current is observed in most chronoamperograms (a). In some cases, it was observed that a current stops flowing after a sharp increase as shown in (b). This current behavior can be described in two ways. Firstly, due to the vigorous hydrogen evolution after DB, conduction spots would be covered partially or totally by the hydrogen bubbles making current flow hard [R1]. Secondly, the unique structure of the conduction spots would reduce net current flow. The current of a recessed ultramicroelectrode is smaller than that of a planar disk ultramicroelectrode [R2]. Moreover, in our experiment, oxide thin film partially covers the recessed Si surface and interrupts mass transport before it is exfoliated mechanically. Therefore, the current decrease subsequent to an initial sharp increase could be explained by the unique structure of the conduction spot and the characteristic of gas evolution reaction. The following current increase is induced by enlarged conduction spot and peeling of the oxide film.

**References**

(R1) German, S. R., Edwards, M. A., Chen, Q., & White, H. S. Laplace Pressure of Individual $H_2$ Nanobubbles from Pressure–Addition Electrochemistry. *Nano Lett.* **16,** 6691–6694 (2016).

(R2) Nogala, W., Velmurugan, J. & Mirkin, M. V. Atomic Force Microscopy of Electrochemical Nanoelectrodes. *Anal. Chem.* **84**, 5192-5197 (2012).

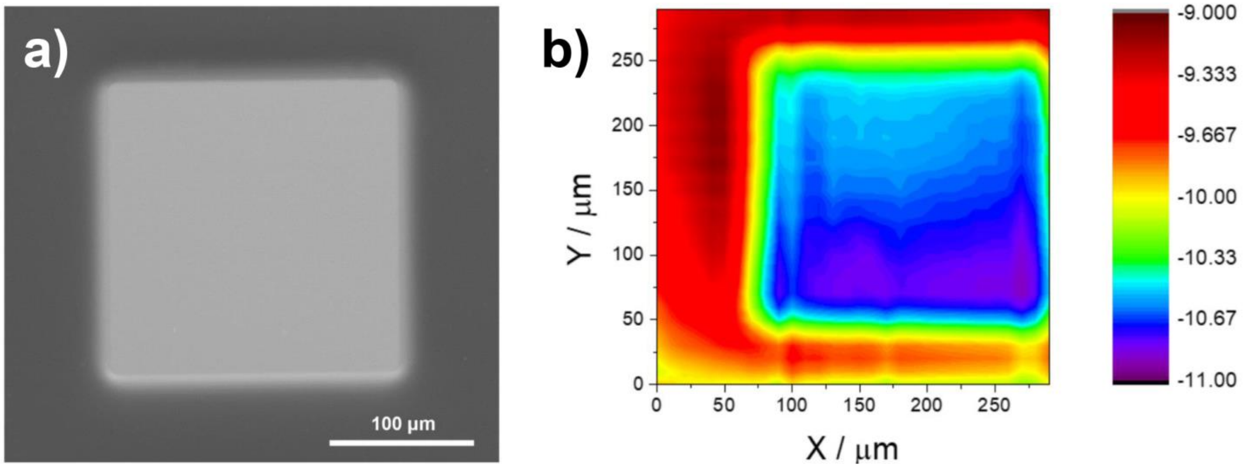


**Figure S4.** (a) Representative SEM and (b) SECM image for 200×200 $\mu m^2$ Si/$SiO_2$ substrate electrode. (b) is obtained in a normal feedback mode, monitoring the $[Ru(NH_3)_6]^{3+}$ reduction current of a tip with a tip potential ($E_{tip}$) held at −0.5 V (vs. SCE) in 10 mM $[Ru(NH_3)_6]Cl_3$ dissolved in 0.1 M PBS (pH 3) without applying any potential to the Si/$SiO_2$ substrate. Distance of tip to substrate = 10 μm. Scan rate = 50 μm $s^{-1}$. The unit of tip current is nA.

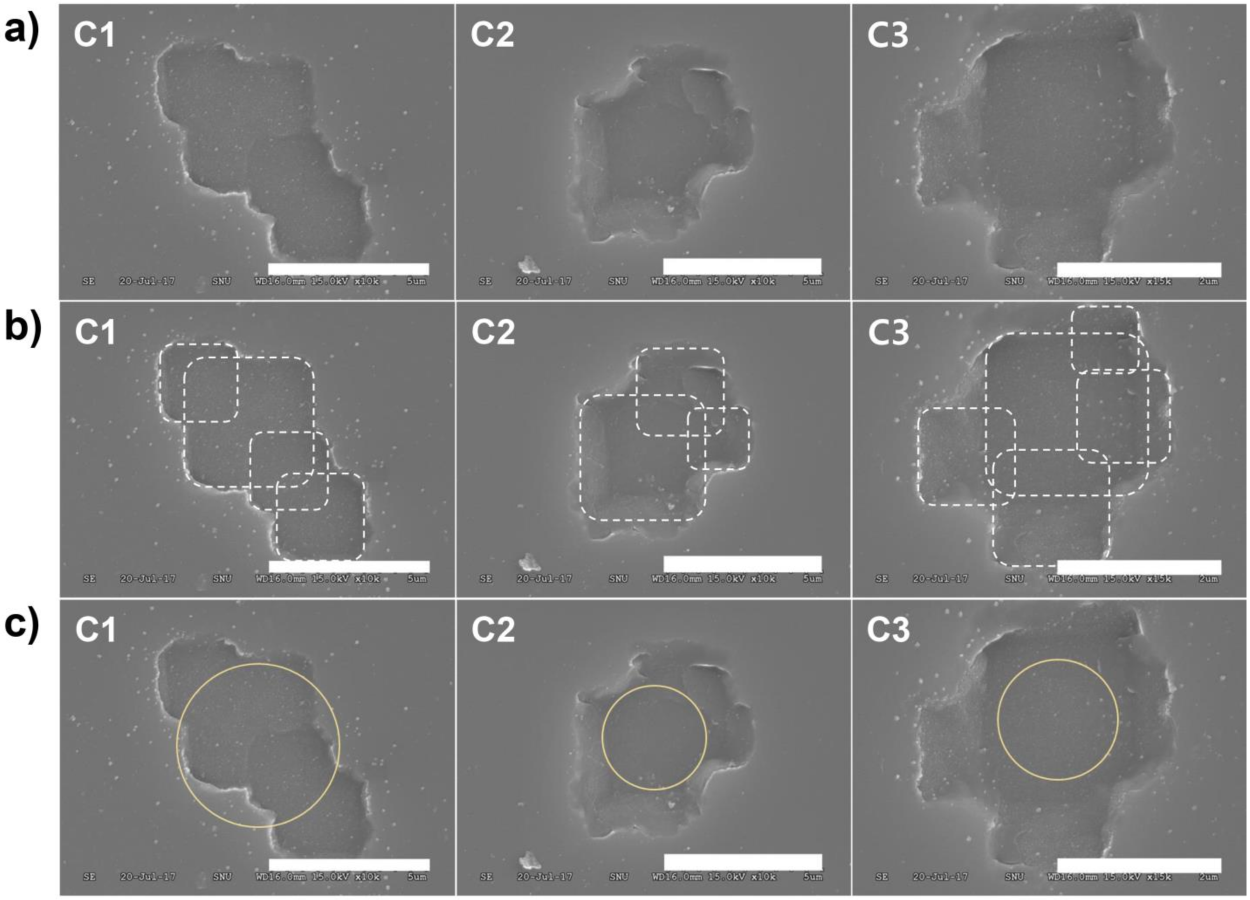


**Figure S5.** (a) Obtained SEM images of C1, C2 and C3 in Figure 3d. (b) Estimated rectangular projected surfaces (white dashed line) from the SEM images and (c) estimated sizes (yellow line) from the SECM images of C1, C2 and C3, assuming the circular geometry of the conduction spots. Scale bar = 5 μm.

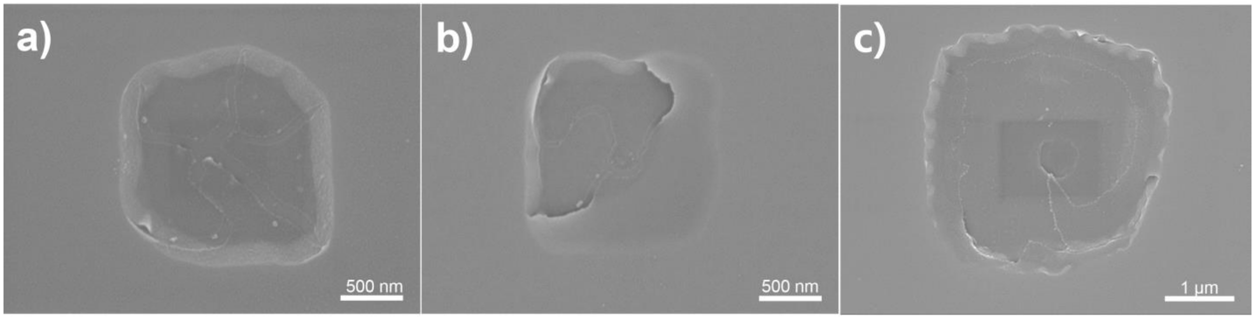


**Figure S6.** SEM images of conduction spots generated at (a and b) ~10 s and (c) ~100 s after a sudden current increase with continuous voltage application of $E_{sub}$ = –4 V in 0.1 M PBS. Tip currents recorded by SECM in 10 mM $[Ru(NH_3)_6]Cl_3$/0.1 M PBS (pH 3) are 60.0 pA for (a) and 33.9 pA for (b) and 0.858 nA for (c), respectively.

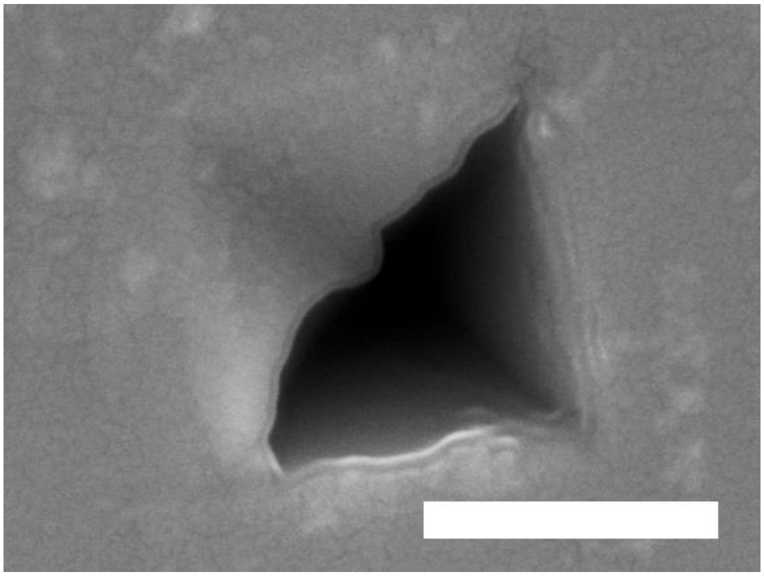

**Figure S7.** A representative SEM image (top view) of an inverted pyramid structure that is partially covered by an oxide film after breakdown. Scale bar = 500 nm.

.

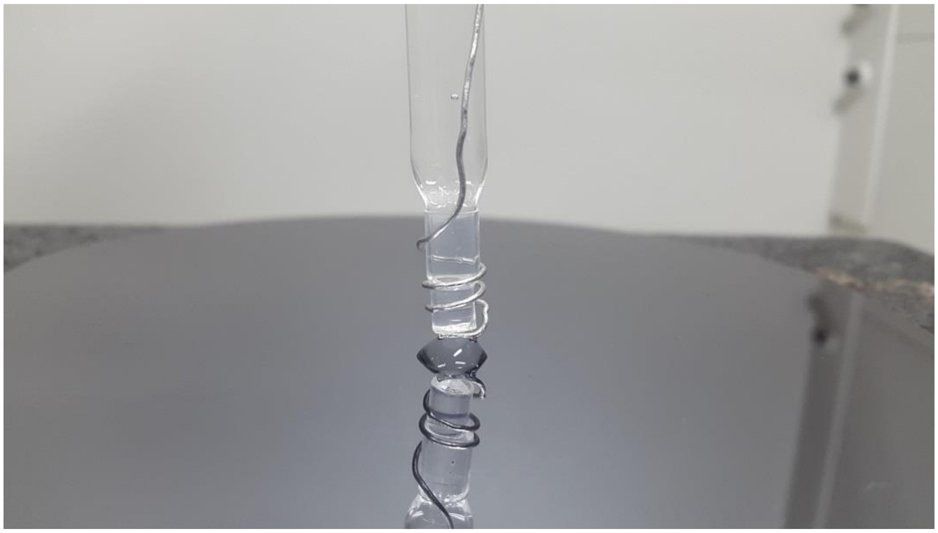

**Figure S8.** An overview picture of experimental setup of Si/$SiO_2$/buffer electrochemical system. The electrochemical measurements were performed by CHI660A potentiostat (CH Instrument, Inc.) in a faraday cage.
